# Supplementary material for: Therapeutic targeting of differentiation-state dependent metabolic vulnerabilities in diffuse midline glioma
Source: Nat Commun. 2024 Oct 17;15:8983. doi: 10.1038/s41467-024-52973-4 (PMC11487135; doi:10.1038/s41467-024-52973-4)
Supplement: Supplementary file 1 — Supplementary Information [file 41467_2024_52973_MOESM1_ESM.pdf]

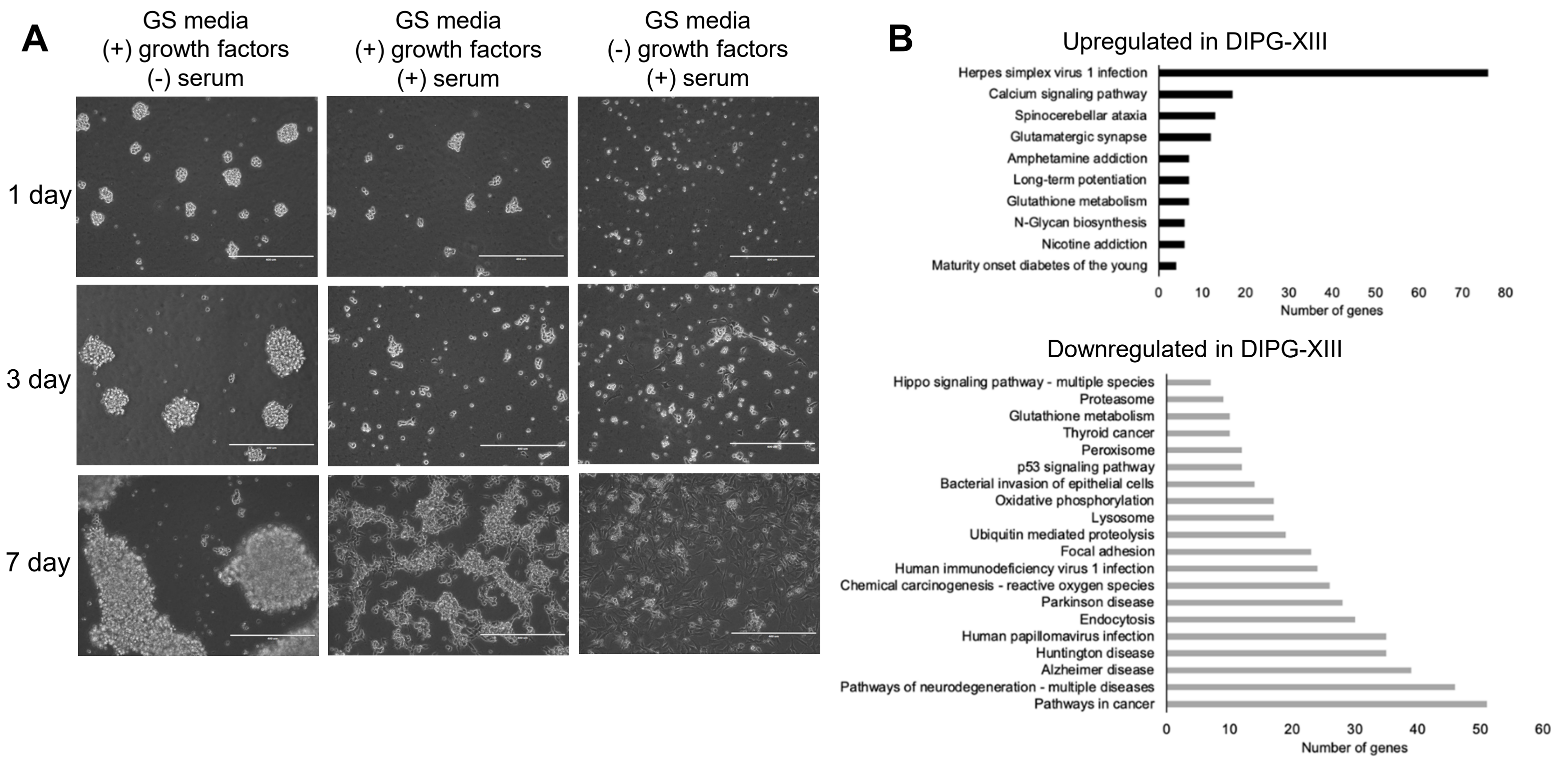

Supplementary Figure 1: Further characterization of the DIPG cultures used in this study. A) Brightfield images (10x) of DIPG-007 gliomaspheres (GS) grown on non-tissue culture-treated surfaces in GS media with and without growth factors and with or without 10% fetal bovine serum (FBS) at the indicated time points; scale bars indicate 400um, n=1 **B)** KEGG pathways uniquely upregulated (top) and down-regulated (bottom) in DIPG-XIII GS relative to DGC.

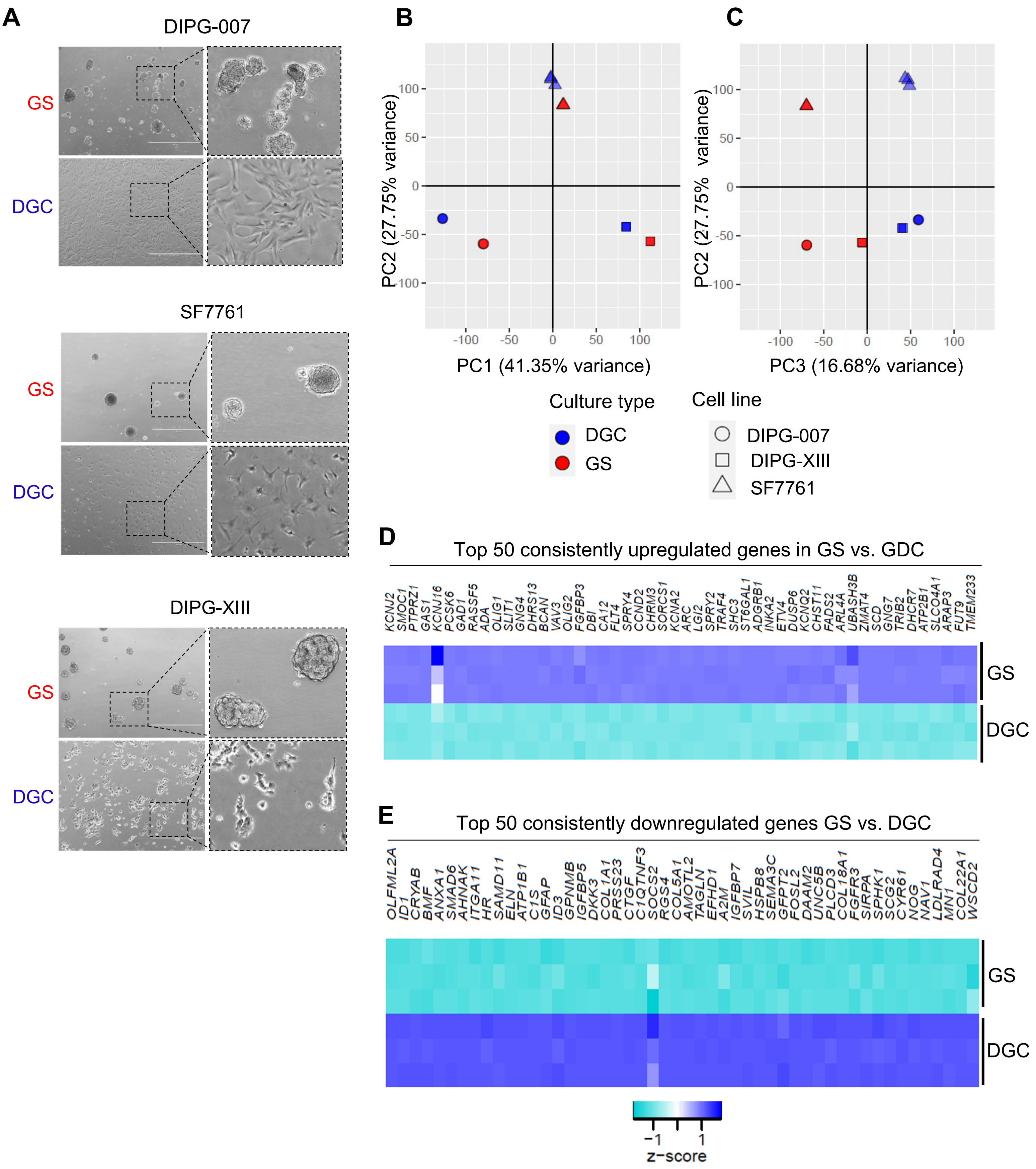

A
DIPG-007
GS
DGC
SF7761
GS
DGC
DIPG-XIII
GS
DGC
B
PC2 (27.75% variance)
100
50
0
-50
-100
-100
-50
0
50
100
PC1 (41.35% variance)
C
PC2 (27.75% variance)
100
50
0
-50
-100
-100
-50
0
50
100
PC3 (16.68% variance)
Culture type
DGC
GS
Cell line
DIPG-007
DIPG-XIII
SF7761
D
Top 50 consistently upregulated genes in GS vs. GDC
KCNJ2 SMOC1 PTPRZ1 GAS1 KCNJ16 PCSK6 GAD1 RASSF5 ADA OLIG1 SLIT1 GNG4 DHRS13 BCAN VAV3 OLIG2 FGFBP3 DBI CA12 FLT4 SPRY4 CCND2 CHRM3 SORCS1 KCNA2 ARC LGI2 SPRY2 TRAF4 SHC3 ST6GAL1 ADGRB1 INKA2 ETV4 DUSP6 KCNQ2 CHST11 FADS2 ARL4A UBASH3B ZMAT4 SCD GNG7 TRIB2 DHCR7 ATP2B1 SLCO4A1 ARAP3 FUT9 TMEM233
GS
DGC
E
Top 50 consistently downregulated genes GS vs. DGC
OLFML2A ID1 CRYAB BMF ANXA1 SMAD6 AHNAK ITGA11 HR SAMD11 ELN ATP1B1 C1S GFAP ID3 GPNMB IGFBP5 DKK3 COL1A1 PRSS23 CTGF C1QTNF3 SOCS2 RGS4 COL5A1 AMOTL2 TAGLN EFHD1 A2M IGFBP7 SVIL HSPB8 SEMA3C GFPT2 FOSL2 DAAM2 UNC5B PLCD3 COL18A1 FGFR3 SIRPA SPHK1 SCG2 CYR61 NOG NAV1 LDLRAD4 MN1 COL22A1 WSCD2
GS
DGC
−1
1
z−score

Supplementary Figure 2: Characterization and transcriptomics analysis of DIPG models. A) Representative images of DIPG-007, SF7761, and DIPG-XIII, showing morphological differences between gliomaspheres (GS) and differentiated glioma cells (DGC); scale bars indicate 1 mm, n=1. **B, C)** Principal component (PC) analysis of RNA-seq data. **D, E)** Heatmaps indicating the top 50 consistently and significantly upregulated **D)** and downregulated **E)** genes common to all three GS vs. DGC lines. The differential expressed genes between the groups were determined using DESeq2 package in R (Wald test, adjusted $p < 0.05$). Heatmaps were generated using the heatmap.2 function in the gplots package in R.For panels A, B and C, red indicates GS; blue, DGC. For panels D and E, blue represents significantly higher expression; green, significantly lower expression.

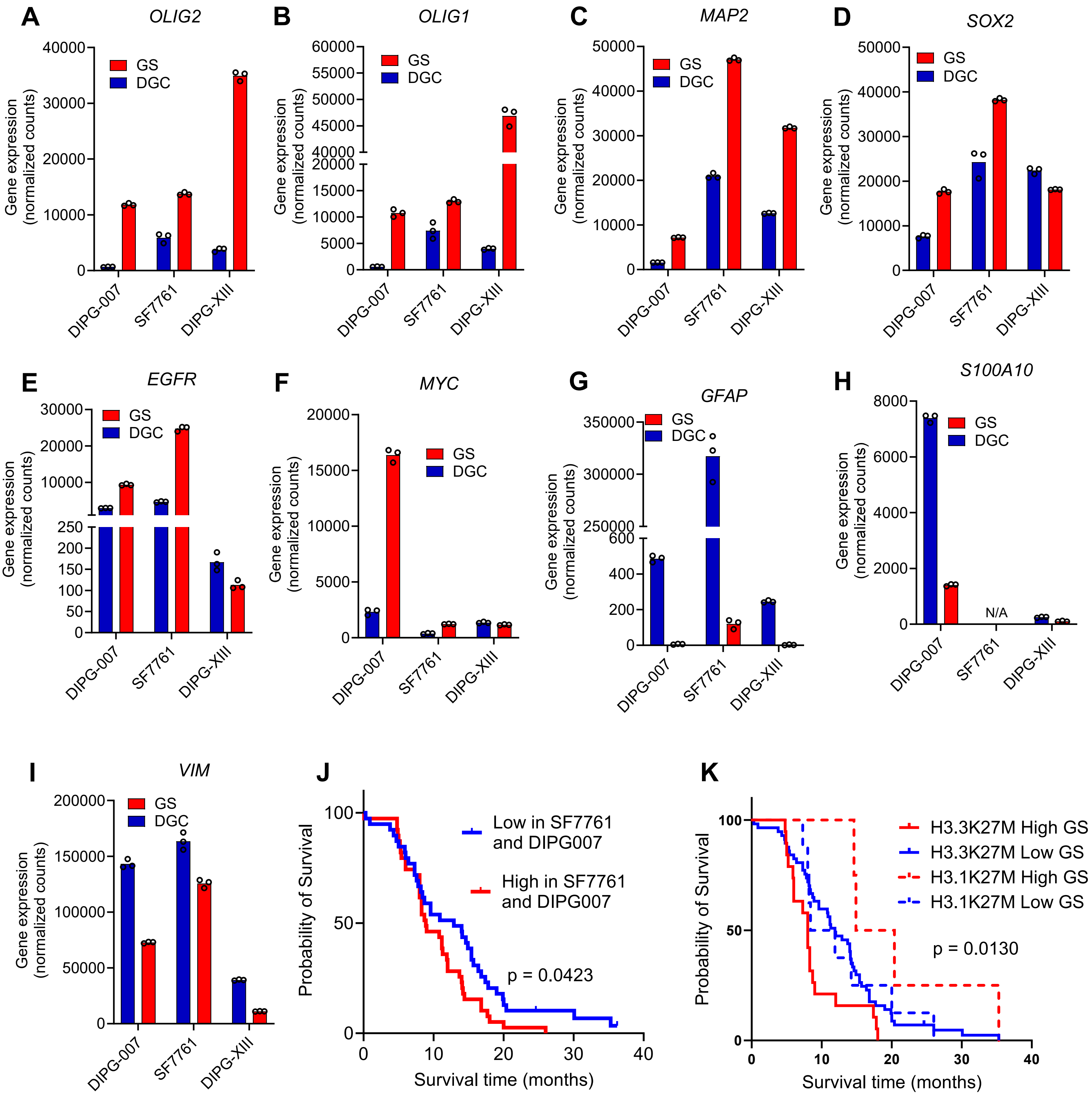

A
OLIG2
B
OLIG1
C
MAP2
D
SOX2
E
EGFR
F
MYC
G
GFAP
H
S100A10
I
VIM
GS
DGC
Gene expression (normalized counts)
DIPG-007
SF7761
DIPG-XIII
N/A
J
Low in SF7761 and DIPG007
High in SF7761 and DIPG007
p = 0.0423
Probability of Survival
Survival time (months)
K
H3.3K27M High GS
H3.3K27M Low GS
H3.1K27M High GS
H3.1K27M Low GS
p = 0.0130

Supplementary Figure 3: Transcriptomics analysis of gene markers in DIPG models. Relative gene expression of DIPG stemness and differentiation markers in gliomaspheres (GS) vs. differentiated glioma cells (DGC) across DIPG-007, SF7761, and DIPG-XIII. Normalized read counts were subjected to the Wald test (two-sided) and p-values were corrected using the Benjamini-Hochberg method. Genes were significant at adjusted p-value < 0.05. RNA-seq data was generated from three technical replicates, n=1. **A)** oligodendrocyte transcription factor 2 (*OLIG2*), **B)** oligodendrocyte transcription factor 1 (*OLIG1*), **C)** microtubule-associated protein 2 (*MAP2*), **D)** SRY-box transcription factor 2 (*SOX2*), **E)** epidermal growth factor receptor (*EGFR*), **F)** *MYC*, **G)** glial fibrillary acidic protein (*GFAP*), **H)** vimentin (*VIM*), **I)** S100 calcium binding protein A10 (*S100A10*). Statistical comparisons including the p-value, p-adjusted value, and shrunkLFC can be found in the Source data file. **J)** Survival analysis of DIPG/diffuse midline gliomas (DMG) patients based on “GS high” versus “GS low” gene signature from SF7761 and DIPG-007 only. Median survival is 8.90 months versus 12.90 months; Log Rank p=0.0423. **K)** Survival analysis of DIPG/DMG patients based on “GS high” versus “GS low” gene signature in H3.3 versus H3.1. Median survival is 8 months (H3.3K27M High GS), 11.9 months (H3.3K27M Low GS), 17.65 months (H3.1K27M High GS), and 10.15 months (H3.1K27M Low GS); Log Rank p=0.0130. For Kaplan-Meier curves in J-K, n = 78; female = 41, male = 28, unknown = 9. For panels A-I, red indicates GS; blue, DGC; for panels J and K, red indicates “GS high;” blue, “GS low.”

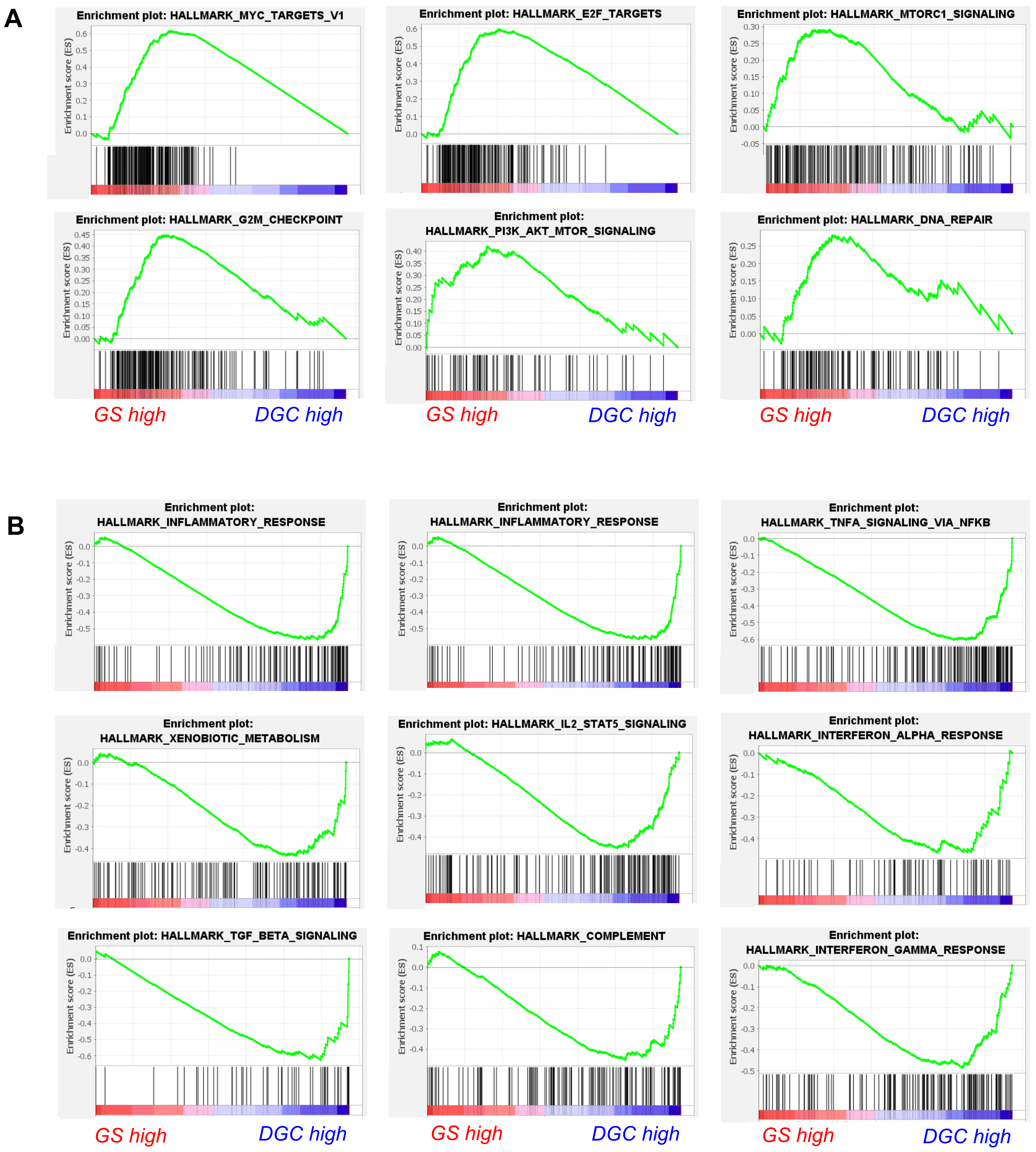
A
Enrichment plot: HALLMARK_MYC_TARGETS_V1
Enrichment plot: HALLMARK_E2F_TARGETS
Enrichment plot: HALLMARK_MTORC1_SIGNALING
Enrichment plot: HALLMARK_G2M_CHECKPOINT
Enrichment plot: HALLMARK_PI3K_AKT_MTOR_SIGNALING
Enrichment plot: HALLMARK_DNA_REPAIR
Enrichment score (ES)
GS high
DGC high
B
Enrichment plot: HALLMARK_INFLAMMATORY_RESPONSE
Enrichment plot: HALLMARK_INFLAMMATORY_RESPONSE
Enrichment plot: HALLMARK_TNFA_SIGNALING_VIA_NFKB
Enrichment plot: HALLMARK_XENOBIOTIC_METABOLISM
Enrichment plot: HALLMARK_IL2_STAT5_SIGNALING
Enrichment plot: HALLMARK_INTERFERON_ALPHA_RESPONSE
Enrichment plot: HALLMARK_TGF_BETA_SIGNALING
Enrichment plot: HALLMARK_COMPLEMENT
Enrichment plot: HALLMARK_INTERFERON_GAMMA_RESPONSE
Enrichment score (ES)
GS high
DGC high

Supplementary Figure 4: Gene set enrichment pathway analysis (GSEA) of DIPG cells. Gene set enrichment analysis (GSEA) indicating pathways that are **A)** upregulated, and **B)** downregulated in DIPG gliomaspheres (GS) vs. differentiated glioma cells (DGC) across all three lines (DIPG-007, SF7761, and DIPG-XIII). GSEA plots show enrichment scores and include values for normalized enrichment score (NES), nominal p value (*P*), and false discovery rate (FDR) q value are derived from GSEA analysis built in within in the software. GSEA plots are derived from RNA-seq analysis generated from three technical replicates, n=1. Red indicates "GS high;" blue, "DGC high."

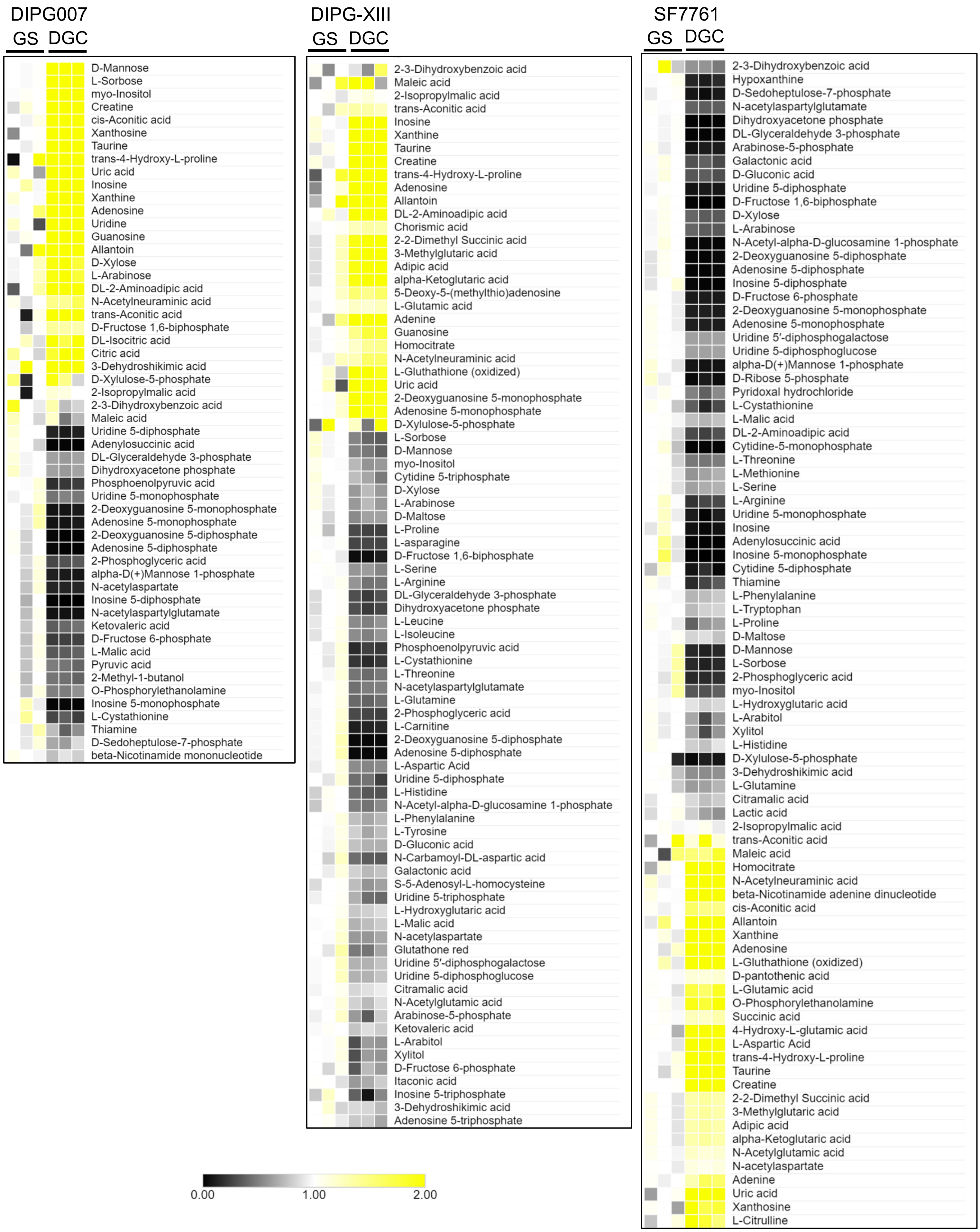
DIPG007
GS
DGC
D-Mannose
L-Sorbose
myo-Inositol
Creatine
cis-Aconitic acid
Xanthosine
Taurine
trans-4-Hydroxy-L-proline
Uric acid
Inosine
Xanthine
Adenosine
Uridine
Guanosine
Allantoin
D-Xylose
L-Arabinose
DL-2-Aminoadipic acid
N-Acetylneuraminic acid
trans-Aconitic acid
D-Fructose 1,6-biphosphate
DL-Isocitric acid
Citric acid
3-Dehydroshikimic acid
D-Xylulose-5-phosphate
2-Isopropylmalic acid
2-3-Dihydroxybenzoic acid
Maleic acid
Uridine 5-diphosphate
Adenylosuccinic acid
DL-Glyceraldehyde 3-phosphate
Dihydroxyacetone phosphate
Phosphoenolpyruvic acid
Uridine 5-monophosphate
2-Deoxyguanosine 5-monophosphate
Adenosine 5-monophosphate
2-Deoxyguanosine 5-diphosphate
Adenosine 5-diphosphate
2-Phosphoglyceric acid
alpha-D(+)Mannose 1-phosphate
N-acetylaspartate
Inosine 5-diphosphate
N-acetylaspartylglutamate
Ketovaleric acid
D-Fructose 6-phosphate
L-Malic acid
Pyruvic acid
2-Methyl-1-butanol
O-Phosphorylethanolamine
Inosine 5-monophosphate
L-Cystathionine
Thiamine
D-Sedoheptulose-7-phosphate
beta-Nicotinamide mononucleotide
DIPG-XIII
GS
DGC
2-3-Dihydroxybenzoic acid
Maleic acid
2-Isopropylmalic acid
trans-Aconitic acid
Inosine
Xanthine
Taurine
Creatine
trans-4-Hydroxy-L-proline
Adenosine
Allantoin
DL-2-Aminoadipic acid
Chorismic acid
2-2-Dimethyl Succinic acid
3-Methylglutaric acid
Adipic acid
alpha-Ketoglutaric acid
5-Deoxy-5-(methylthio)adenosine
L-Glutamic acid
Adenine
Guanosine
Homocitrate
N-Acetylneuraminic acid
L-Gluthathione (oxidized)
Uric acid
2-Deoxyguanosine 5-monophosphate
Adenosine 5-monophosphate
D-Xylulose-5-phosphate
L-Sorbose
D-Mannose
myo-Inositol
Cytidine 5-triphosphate
D-Xylose
L-Arabinose
D-Maltose
L-Proline
L-asparagine
D-Fructose 1,6-biphosphate
L-Serine
L-Arginine
DL-Glyceraldehyde 3-phosphate
Dihydroxyacetone phosphate
L-Leucine
L-Isoleucine
Phosphoenolpyruvic acid
L-Cystathionine
L-Threonine
N-acetylaspartylglutamate
L-Glutamine
2-Phosphoglyceric acid
L-Carnitine
2-Deoxyguanosine 5-diphosphate
Adenosine 5-diphosphate
L-Aspartic Acid
Uridine 5-diphosphate
L-Histidine
N-Acetyl-alpha-D-glucosamine 1-phosphate
L-Phenylalanine
L-Tyrosine
D-Gluconic acid
N-Carbamoyl-DL-aspartic acid
Galactonic acid
S-5-Adenosyl-L-homocysteine
Uridine 5-triphosphate
L-Hydroxyglutaric acid
L-Malic acid
N-acetylaspartate
Glutathone red
Uridine 5′-diphosphogalactose
Uridine 5-diphosphoglucose
Citramalic acid
N-Acetylglutamic acid
Arabinose-5-phosphate
Ketovaleric acid
L-Arabitol
Xylitol
D-Fructose 6-phosphate
Itaconic acid
Inosine 5-triphosphate
3-Dehydroshikimic acid
Adenosine 5-triphosphate
SF7761
GS
DGC
2-3-Dihydroxybenzoic acid
Hypoxanthine
D-Sedoheptulose-7-phosphate
N-acetylaspartylglutamate
Dihydroxyacetone phosphate
DL-Glyceraldehyde 3-phosphate
Arabinose-5-phosphate
Galactonic acid
D-Gluconic acid
Uridine 5-diphosphate
D-Fructose 1,6-biphosphate
D-Xylose
L-Arabinose
N-Acetyl-alpha-D-glucosamine 1-phosphate
2-Deoxyguanosine 5-diphosphate
Adenosine 5-diphosphate
Inosine 5-diphosphate
D-Fructose 6-phosphate
2-Deoxyguanosine 5-monophosphate
Adenosine 5-monophosphate
Uridine 5′-diphosphogalactose
Uridine 5-diphosphoglucose
alpha-D(+)Mannose 1-phosphate
D-Ribose 5-phosphate
Pyridoxal hydrochloride
L-Cystathionine
L-Malic acid
DL-2-Aminoadipic acid
Cytidine-5-monophosphate
L-Threonine
L-Methionine
L-Serine
L-Arginine
Uridine 5-monophosphate
Inosine
Adenylosuccinic acid
Inosine 5-monophosphate
Cytidine 5-diphosphate
Thiamine
L-Phenylalanine
L-Tryptophan
L-Proline
D-Maltose
D-Mannose
L-Sorbose
2-Phosphoglyceric acid
myo-Inositol
L-Hydroxyglutaric acid
L-Arabitol
Xylitol
L-Histidine
D-Xylulose-5-phosphate
3-Dehydroshikimic acid
L-Glutamine
Citramalic acid
Lactic acid
2-Isopropylmalic acid
trans-Aconitic acid
Maleic acid
Homocitrate
N-Acetylneuraminic acid
beta-Nicotinamide adenine dinucleotide
cis-Aconitic acid
Allantoin
Xanthine
Adenosine
L-Gluthathione (oxidized)
D-pantothenic acid
L-Glutamic acid
O-Phosphorylethanolamine
Succinic acid
4-Hydroxy-L-glutamic acid
L-Aspartic Acid
trans-4-Hydroxy-L-proline
Taurine
Creatine
2-2-Dimethyl Succinic acid
3-Methylglutaric acid
Adipic acid
alpha-Ketoglutaric acid
N-Acetylglutamic acid
N-acetylaspartate
Adenine
Uric acid
Xanthosine
L-Citrulline
0.00
1.00
2.00

Supplementary Figure 5: Steady-state metabolomics profiling of DIPG cells. Heatmaps of each individual DIPG cell line pair (DIPG-007, SF7761, and DIPG-XIII) showing significantly altered ($p < 0.01$) and differential abundance of metabolites; metabolites presented are selected from among the 223 detected (see Supplemental Data Table 2). Statistically significant differential metabolites were evaluated using t-test with two-tailed distribution and two-sample unequal variance for independently prepared samples in triplicate. Columns represent independently prepared replicates; rows represent median normalized metabolites within each pair of differentiation models. Each individual cell line was normalized to its gliomasphere (GS) metabolic abundance, and, therefore, yellow indicates highly abundant metabolites while black indicates highly depleted metabolites in differentiated glioma cells (DGC) as compared to GS. Metabolomic profiling data were generated from the average of three replicates samples prepared for 1 experiment.

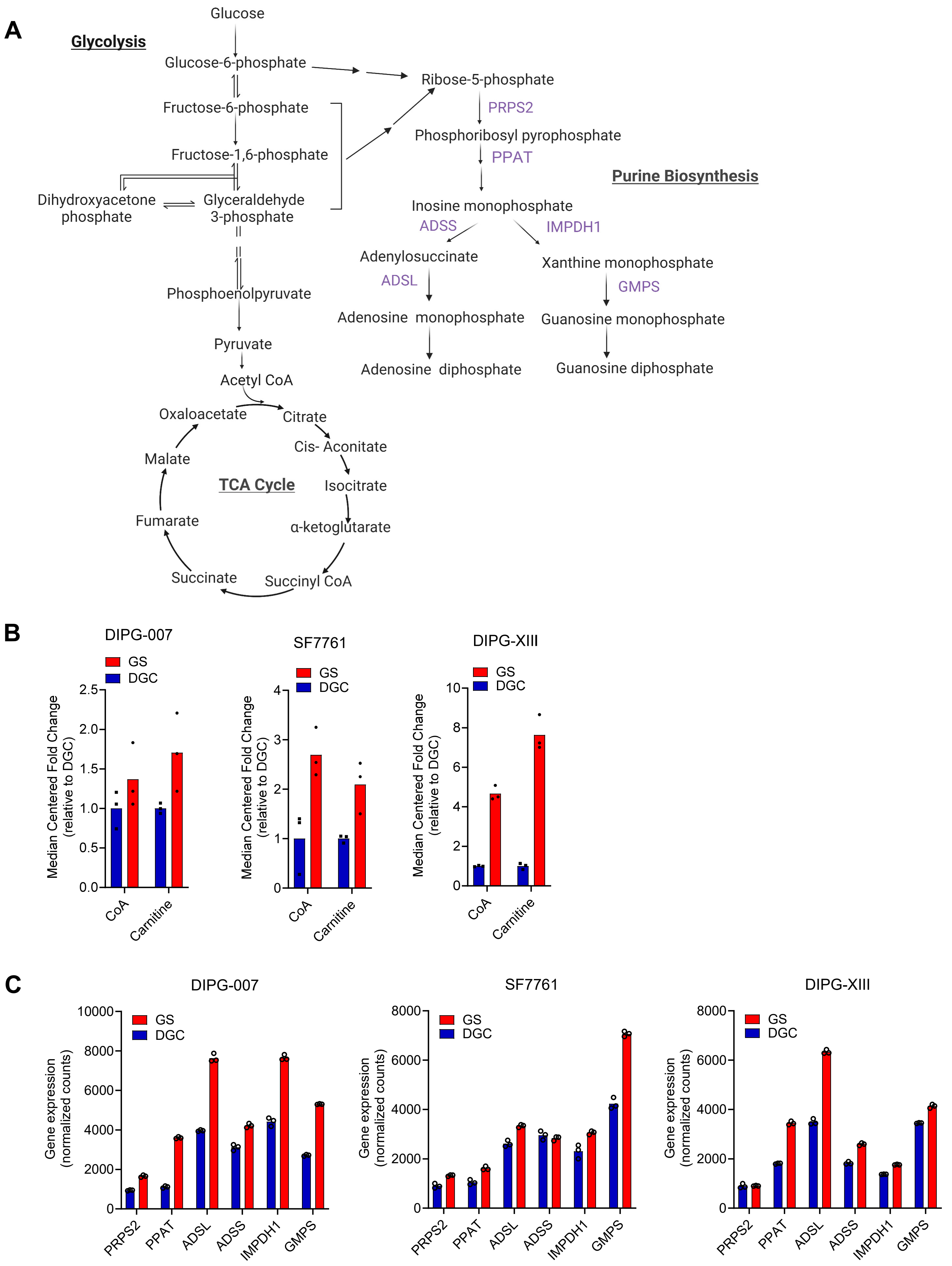
A
Glycolysis
Glucose
Glucose-6-phosphate
Fructose-6-phosphate
Fructose-1,6-phosphate
Dihydroxyacetone phosphate
Glyceraldehyde 3-phosphate
Phosphoenolpyruvate
Pyruvate
Acetyl CoA
Ribose-5-phosphate
PRPS2
Phosphoribosyl pyrophosphate
PPAT
Purine Biosynthesis
Inosine monophosphate
ADSS
IMPDH1
Adenylosuccinate
Xanthine monophosphate
ADSL
GMPS
Adenosine monophosphate
Guanosine monophosphate
Adenosine diphosphate
Guanosine diphosphate
Oxaloacetate
Citrate
Cis- Aconitate
Malate
TCA Cycle
Isocitrate
Fumarate
α-ketoglutarate
Succinate
Succinyl CoA
B
DIPG-007
SF7761
DIPG-XIII
GS
DGC
Median Centered Fold Change (relative to DGC)
CoA
Carnitine
C
DIPG-007
SF7761
DIPG-XIII
GS
DGC
Gene expression (normalized counts)
PRPS2
PPAT
ADSL
ADSS
IMPDH1
GMPS

Supplementary Figure 6: Metabolic features of DIPG cells. A) Schematic of central metabolic pathways highlighting connections in glycolysis, TCA cycle, and purine biosynthetic pathways. **B)** Levels of CoA and Carnitine in DIPG-007, SF7761, and DIPG-XIII gliomasphere (GS) vs. differentiated glioma cell (DGC) populations. Metabolite levels expressed as median-centered fold change of GS relative to DGC across the three lines from three technical replicates, n=1. **C)** Relative expression of genes (bulk RNA-seq data) encoding enzymes involved in purine biosynthesis in DIPG-007, SF7761, and DIPG-XIII GS and DGC counterparts. Data were generated from three technical replicates, n=1. Normalized read counts were subjected to the Wald test (two-sided). PRPS2, phosphoribosyl pyrophosphate synthetase 2; PPAT, phosphoribosyl pyrophosphate amidotransferase; ADSL, adenylosuccinate lyase; ADSS, adenylosuccinate synthase; IMPDH1, inosine monophosphate dehydrogenase 1; GMPS, guanine monophosphate synthase. For panels B and C, red indicates GS; blue, DGC.

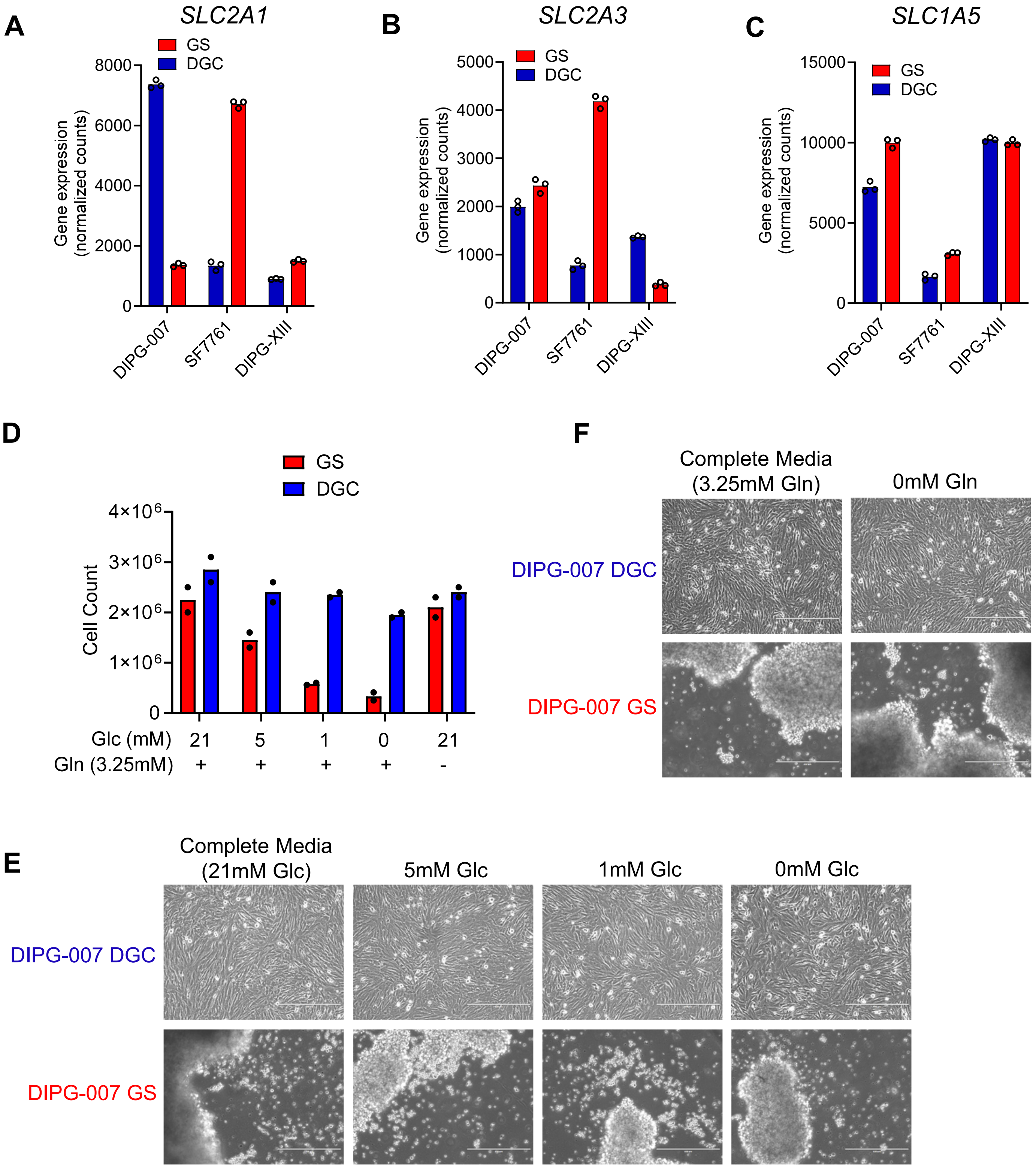
A
SLC2A1
GS
DGC
Gene expression (normalized counts)
8000
6000
4000
2000
0
DIPG-007
SF7761
DIPG-XIII
B
SLC2A3
GS
DGC
Gene expression (normalized counts)
5000
4000
3000
2000
1000
0
DIPG-007
SF7761
DIPG-XIII
C
SLC1A5
GS
DGC
Gene expression (normalized counts)
15000
10000
5000
0
DIPG-007
SF7761
DIPG-XIII
D
GS
DGC
Cell Count
4×10^6
3×10^6
2×10^6
1×10^6
0
Glc (mM) 21 5 1 0 21
Gln (3.25mM) + + + + -
F
Complete Media (3.25mM Gln)
0mM Gln
DIPG-007 DGC
DIPG-007 GS
E
Complete Media (21mM Glc)
5mM Glc
1mM Glc
0mM Glc
DIPG-007 DGC
DIPG-007 GS

Supplementary Figure 7: Glucose and glutamine utilization in DIPG gliomaspheres (GS) vs differentiated glioma cells (DGC). A-C) Relative expression of glucose and glutamine transporters in GS vs. DGC across DIPG-007, SF7761, and DIPG-XIII cell lines. Gene expression normalized counts for solute carrier family 2 member 1 (*SLC2A1*), *SLC2A3* and *SLC1A5* were plotted from bulk transcriptomics analyses of three technical replicates, n=1. **D)** Proliferation of DIPG-007 cells in media with varying glucose (Glc) concentrations and in media with or without glutamine (Gln, 3.25mM) for 7 days was assessed using the Countess II FL Automated Cell Counter (Invitrogen). Results are the average cell count of 2 independent wells, n=1. **E,F)** Representative brightfield images (10x) of the data in (**D**) from two independent wells, n=1; scale bars indicate 400μm. For all panels, red indicates GS; blue, DGC.

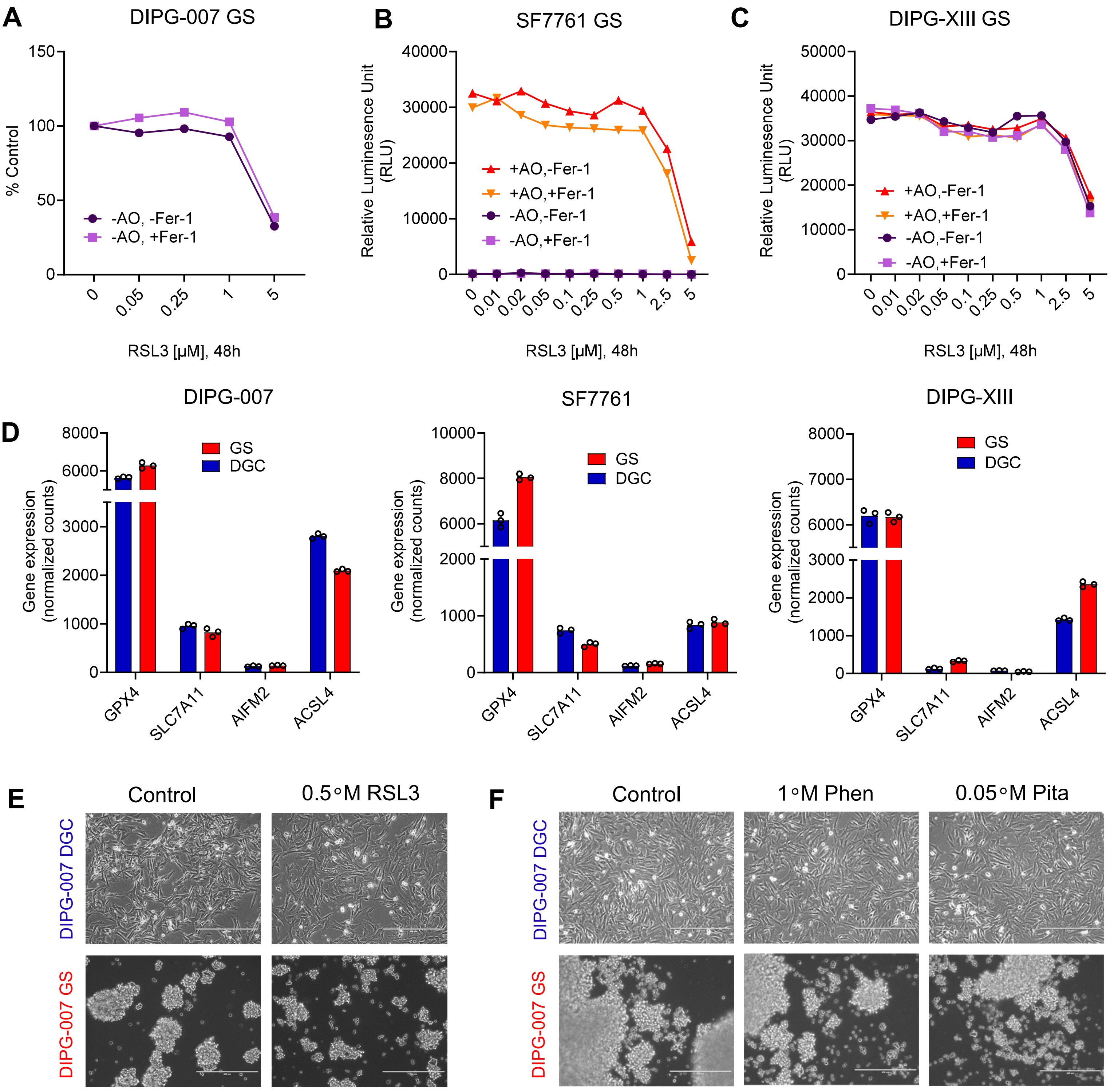
A
DIPG-007 GS
% Control
-AO, -Fer-1
-AO, +Fer-1
RSL3 [µM], 48h
B
SF7761 GS
Relative Luminesence Unit (RLU)
+AO,-Fer-1
+AO,+Fer-1
-AO,-Fer-1
-AO,+Fer-1
RSL3 [µM], 48h
C
DIPG-XIII GS
Relative Luminesence Unit (RLU)
+AO,-Fer-1
+AO,+Fer-1
-AO,-Fer-1
-AO,+Fer-1
RSL3 [µM], 48h
D
DIPG-007
SF7761
DIPG-XIII
Gene expression (normalized counts)
GS
DGC
GPX4
SLC7A11
AIFM2
ACSL4
E
Control
0.5∘M RSL3
DIPG-007 DGC
DIPG-007 GS
F
Control
1∘M Phen
0.05∘M Pita
DIPG-007 DGC
DIPG-007 GS

Supplementary Figure 8: Ferroptosis properties and differentiation state in DIPG gliomaspheres (GS) and differentiated glioma cells (DGC). RSL3 dose response in the presence or absence of Ferrostatin-1 (Fer-1) in **A)** DIPG-007, **B)** SF7761, and **C)** DIPG-XIII GS cells with (+AO) or without (-AO) antioxidants in the B-27 media supplement. Cell viability was assayed at 3 days post-treatment using the Cell Titer-Glo 3D reagent. Results expressed as percent of control (A) or as relative luminescence units (B,C) representing mean of three technical replicates, n=1. **D)** Relative expression of canonical ferroptosis regulators in GS vs. DGC across DIPG-007, SF7761, and DIPG-XIII. Gene expression normalized counts plotted from the bulk transcriptomics analyses using three technical replicates, n=1. Brightfield images (10x) of DIPG-007 DGC and GS grown in the continuous presence of low-dose **E)** RSL3 (0.5 μM), **F)** Phenformin (Phen, 1 μM) or Pitavastatin (Pita, 0.05 μM) for 7 days to assess differentiation morphology in two independent wells, n=1; scale bars indicate 400um. GPX4, glutathione peroxidase 4; SLC7A11, solute carrier family 7 member 11; AIFM2, apoptosis inducing factor mitochondria associated 2; ACSL4, acyl-CoA synthetase long chain family member. For panels D-F, red indicates GS; blue, DGC.

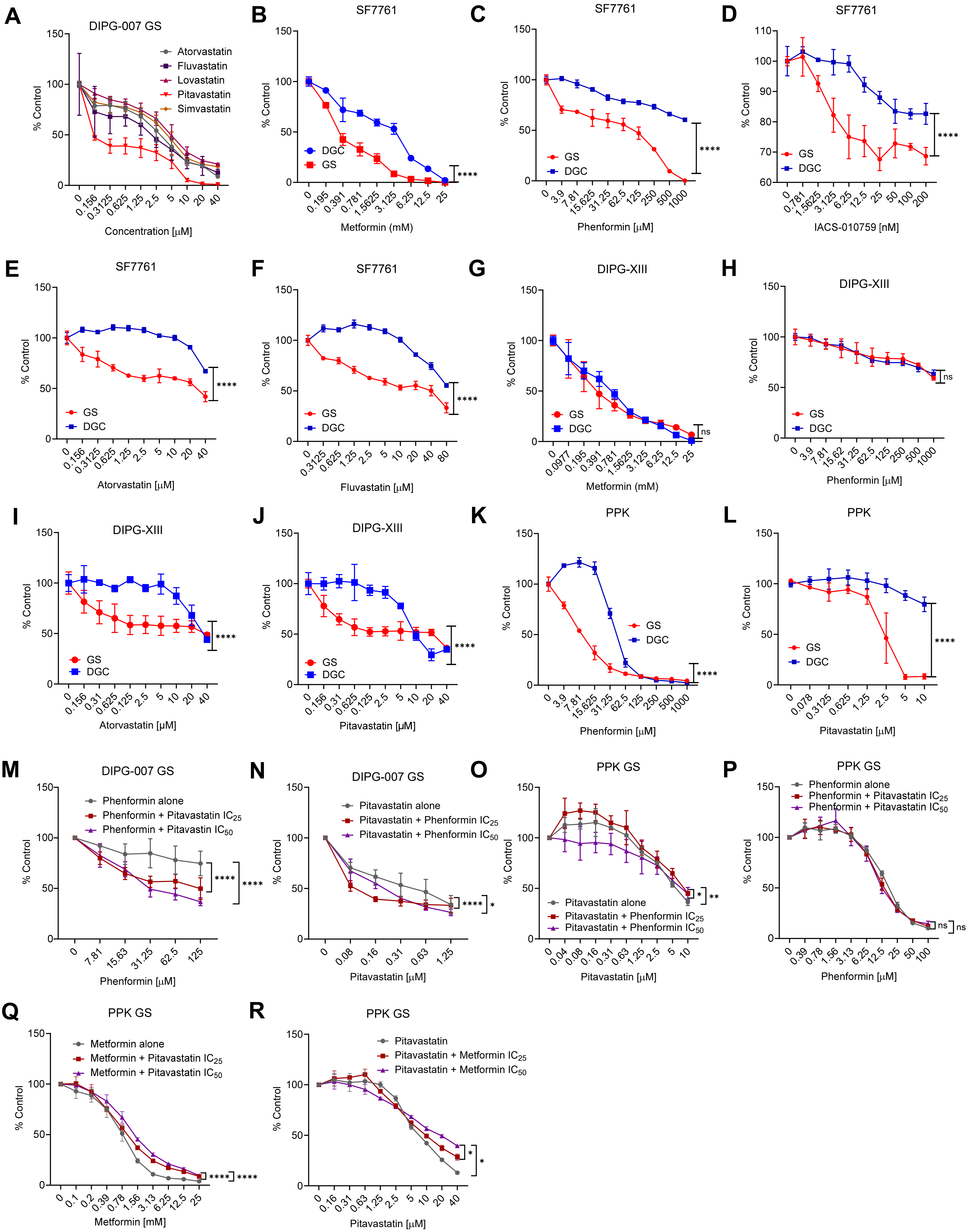

A
DIPG-007 GS
% Control
Atorvastatin
Fluvastatin
Lovastatin
Pitavastatin
Simvastatin
Concentration [μM]
B
SF7761
DGC
GS
****
Metformin (mM)
C
SF7761
GS
DGC
****
Phenformin [μM]
D
SF7761
GS
DGC
****
IACS-010759 [nM]
E
SF7761
GS
DGC
****
Atorvastatin [μM]
F
SF7761
GS
DGC
****
Fluvastatin [μM]
G
DIPG-XIII
GS
DGC
ns
Metformin (mM)
H
DIPG-XIII
GS
DGC
ns
Phenformin [μM]
I
DIPG-XIII
GS
DGC
****
Atorvastatin [μM]
J
DIPG-XIII
GS
DGC
****
Pitavastatin [μM]
K
PPK
GS
DGC
****
Phenformin [μM]
L
PPK
GS
DGC
****
Pitavastatin [μM]
M
DIPG-007 GS
Phenformin alone
Phenformin + Pitavastatin IC_{25}
Phenformin + Pitavastin IC_{50}
**** ****
Phenformin [μM]
N
DIPG-007 GS
Pitavastatin alone
Pitavastatin + Phenformin IC_{25}
Pitavastatin + Phenformin IC_{50}
**** *
Pitavastatin [μM]
O
PPK GS
Pitavastatin alone
Pitavastatin + Phenformin IC_{25}
Pitavastatin + Phenformin IC_{50}
* **
Pitavastatin [μM]
P
PPK GS
Phenformin alone
Phenformin + Pitavastatin IC_{25}
Phenformin + Pitavastatin IC_{50}
ns ns
Phenformin [μM]
Q
PPK GS
Metformin alone
Metformin + Pitavastatin IC_{25}
Metformin + Pitavastatin IC_{50}
**** ****
Metformin [mM]
R
PPK GS
Pitavastatin
Pitavastatin + Metformin IC_{25}
Pitavastatin + Metformin IC_{50}
* *
Pitavastatin [μM]

Supplementary Figure 9: *In vitro* targeting of DIPG cells with OXPHOS inhibitors and statins. A) Efficacy comparison of various clinical statins (Atorvastatin, Fluvastatin, Lovastatin, Pitavastatin, and Simvastatin) in DIPG-007 gliomaspheres (GS). **B-F)** Dose response curves of SF7761 GS and differentiated glioma cells (DGC) treated with **B)** Metformin, **C)** Phenformin, **D)** IACS-010759, **E)** Atorvastatin, and **F)** Fluvastatin. **G-J)** Dose response curves of DIPG-XIII GS and DGC treated with **G)** Metformin, **H)** Phenformin, **I)** Atorvastatin, and **J)** Pitavastatin. **K,L)** Dose response curves of PPK (murine H3K27M) GS and DGC treated with **K)** Phenformin, and **L)** Pitavastatin. **M-R)** Combination drug treatment studies. **M)** DIPG-007 GS co-treated with IC_{25} or IC_{50} of Pitavastatin, relative to vehicle control, across a dose response of Phenformin or **N)** co-treated with the IC_{25} or IC_{50} of Phenformin, relative to vehicle control, across a dose response of Pitavastatin. **O)** PPK GS co-treated with the IC_{25} or IC_{50} of Phenformin, relative to vehicle control, across a dose response of Pitavastatin or **P)** co-treated with the IC_{25} or IC_{50} of Pitavastatin, relative to vehicle control, across a dose response of Phenformin. **Q)** PPK GS co-treated with the IC_{25} or IC_{50} of Pitavastatin, relative to vehicle control, across a dose response of Metformin or **R)** co-treated with the IC_{25} or IC_{50} of Metformin, relative to vehicle control, across a dose response of Pitavastatin. For all cell lines and treatments, cell viability was assayed using Cell Titer-Glo 2.0 (DGC) or 3D (GS) at 3 days or 7 days (Metformin) post-treatment. All results are expressed as percent of control and mean ± SD from three biological replicates; ns = not significant, * $p < 0.05$; ** $p < 0.01$; *** $p < 0.001$; **** $p < 0.0001$; by area under proliferation curve test followed by two-tailed Student's t-test (A-L) or One-way ANOVA test (M-R). For panels B-L, red indicates GS; blue, DGC.

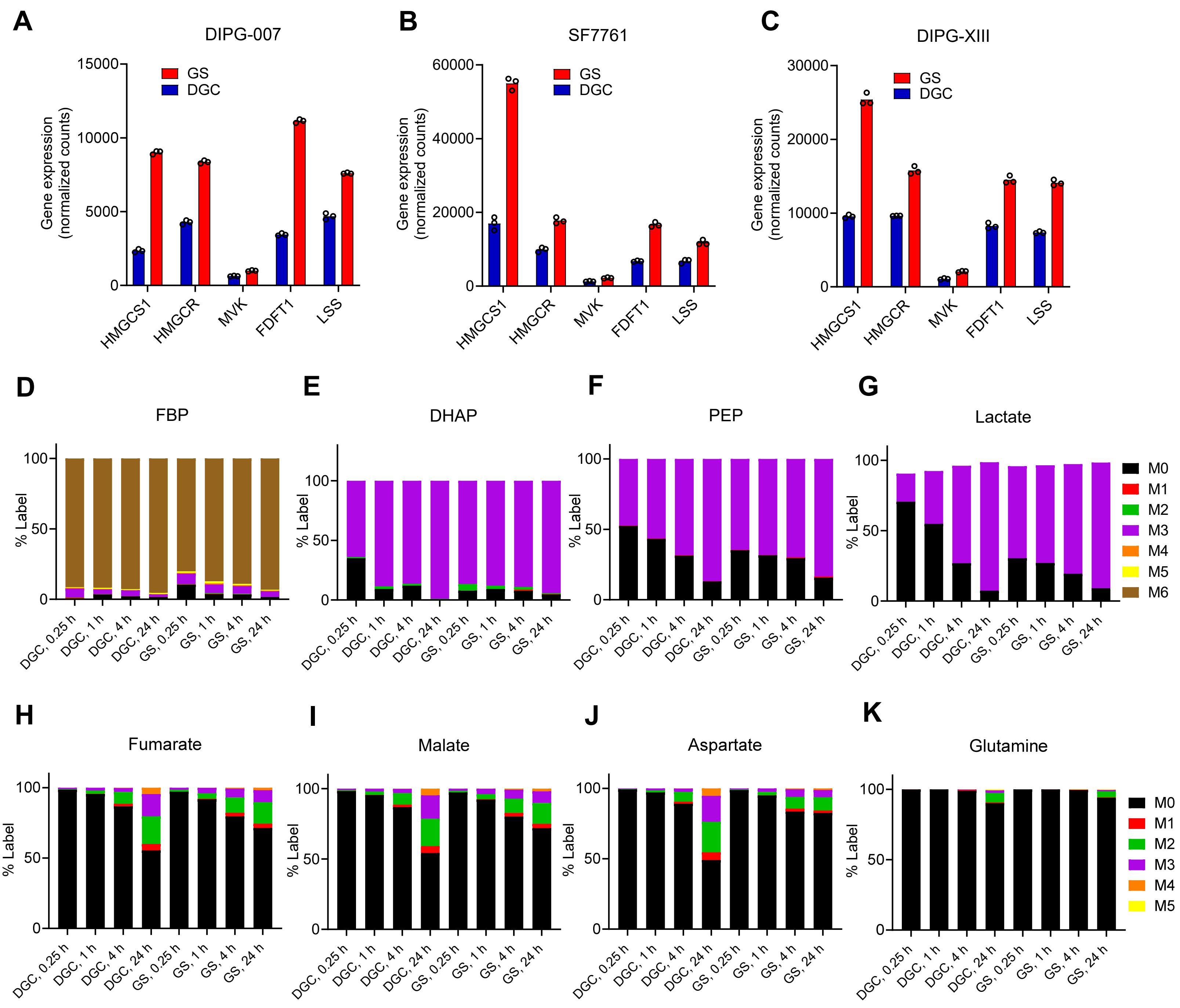

A
DIPG-007
B
SF7761
C
DIPG-XIII
GS
DGC
Gene expression (normalized counts)
HMGCS1
HMGCR
MVK
FDFT1
LSS
D
FBP
E
DHAP
F
PEP
G
Lactate
H
Fumarate
I
Malate
J
Aspartate
K
Glutamine
% Label
DGC, 0.25 h
DGC, 1 h
DGC, 4 h
DGC, 24 h
GS, 0.25 h
GS, 1 h
GS, 4 h
GS, 24 h
M0
M1
M2
M3
M4
M5
M6

Supplementary Figure 10: Sterol biosynthetic pathway gene markers and glucose metabolism in gliomaspheres (GS) and differentiated glioma cells (**DGC**). **A-C)** Relative expression of genes encoding enzymes involved in cholesterol biosynthesis in DIPG-007, SF7761, and DIPG-XIII GS and DGC. Gene expression normalized counts plotted from the bulk transcriptomics analysis with three technical replicates, n=1. **D-K)** Metabolomics-based assessment of glucose-derived carbon entry into downstream metabolism in DIPG-007 DGC or GS at the indicated timepoints. Metabolites are presented for **D-G)** glycolysis, **H,I)** the TCA cycle, and **J,K)** non-essential amino acids. Enrichment is presented as mass + the number of carbons labeled in the metabolite, m+n. Data are presented as the fractional enrichment of the pool. Each colored, stacked bar represents each of the isotopologues and the bar height within each group is determined by the mean calculated from three technical replicates, n=1. HMGCS1, 3-hydroxy-3-methylglutaryl-CoA synthase 1; HMGCR, HMG-CoA reductase; MVK, mevalonate kinase; FDFT1, farnesyl-diphosphate farnesyltransferase 1; LSS, lanosterol synthase; FBP, fructose-1,6-bisphosphate; DHAP, dihydroxyacetone phosphate; PEP, phosphoenolpyruvate. For panels A-C, red indicates GS; blue, DGC.

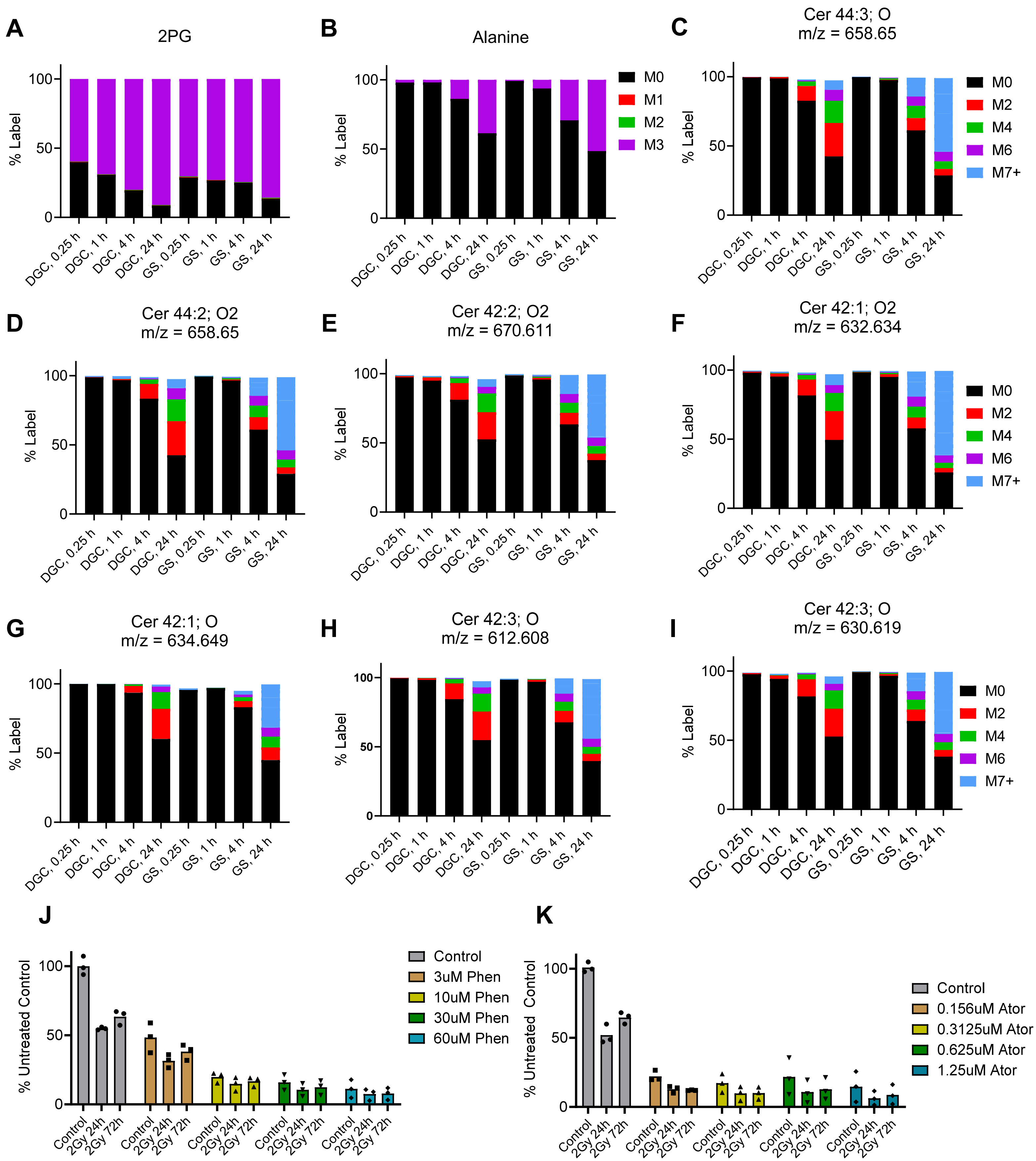

A
2PG
B
Alanine
C
Cer 44:3; O
m/z = 658.65
D
Cer 44:2; O2
m/z = 658.65
E
Cer 42:2; O2
m/z = 670.611
F
Cer 42:1; O2
m/z = 632.634
G
Cer 42:1; O
m/z = 634.649
H
Cer 42:3; O
m/z = 612.608
I
Cer 42:3; O
m/z = 630.619
% Label
M0
M1
M2
M3
M4
M6
M7+
DGC, 0.25 h
DGC, 1 h
DGC, 4 h
DGC, 24 h
GS, 0.25 h
GS, 1 h
GS, 4 h
GS, 24 h
J
% Untreated Control
Control
3uM Phen
10uM Phen
30uM Phen
60uM Phen
2Gy 24h
2Gy 72h
K
% Untreated Control
Control
0.156uM Ator
0.3125uM Ator
0.625uM Ator
1.25uM Ator

Supplementary Figure 11: Glucose metabolism and radiation combination studies. **A,B)** Metabolomics and **C-I)** lipidomics-based assessment of glucose-derived carbon entry into downstream metabolism in DIPG-007 differentiated glioma cells (DGC) or gliomaspheres (GS) at the indicated timepoints. Metabolites are presented for **A,B)** glycolysis and **C-I)** ceramides generated via *de novo* lipid biosynthesis. Enrichment is presented as mass + the number of carbons labeled in the metabolite, m+n. Data are presented as the fractional enrichment of the pool. Each colored, stacked bar represents each of the isotopologues and the bar height within each group is determined by the mean calculated from three technical replicates, n=1. 2PG, 2-phosphoglycerate; Cer (XX:X;OX), ceramide (carbons:unsaturation;oxygens); m/z, mass/charge. **J,K)** DIPG-007 GS treated with **J)** Phenformin (Phen) or **K)** Atorvastatin (Ator) with or without 2 gray (Gy) radiation treatment at 24 or 72 hours (h). Cell viability was assessed after 7 days using Cell Titer-Glo 3D. Results are expressed as percent of control from three technical replicates, n=1.

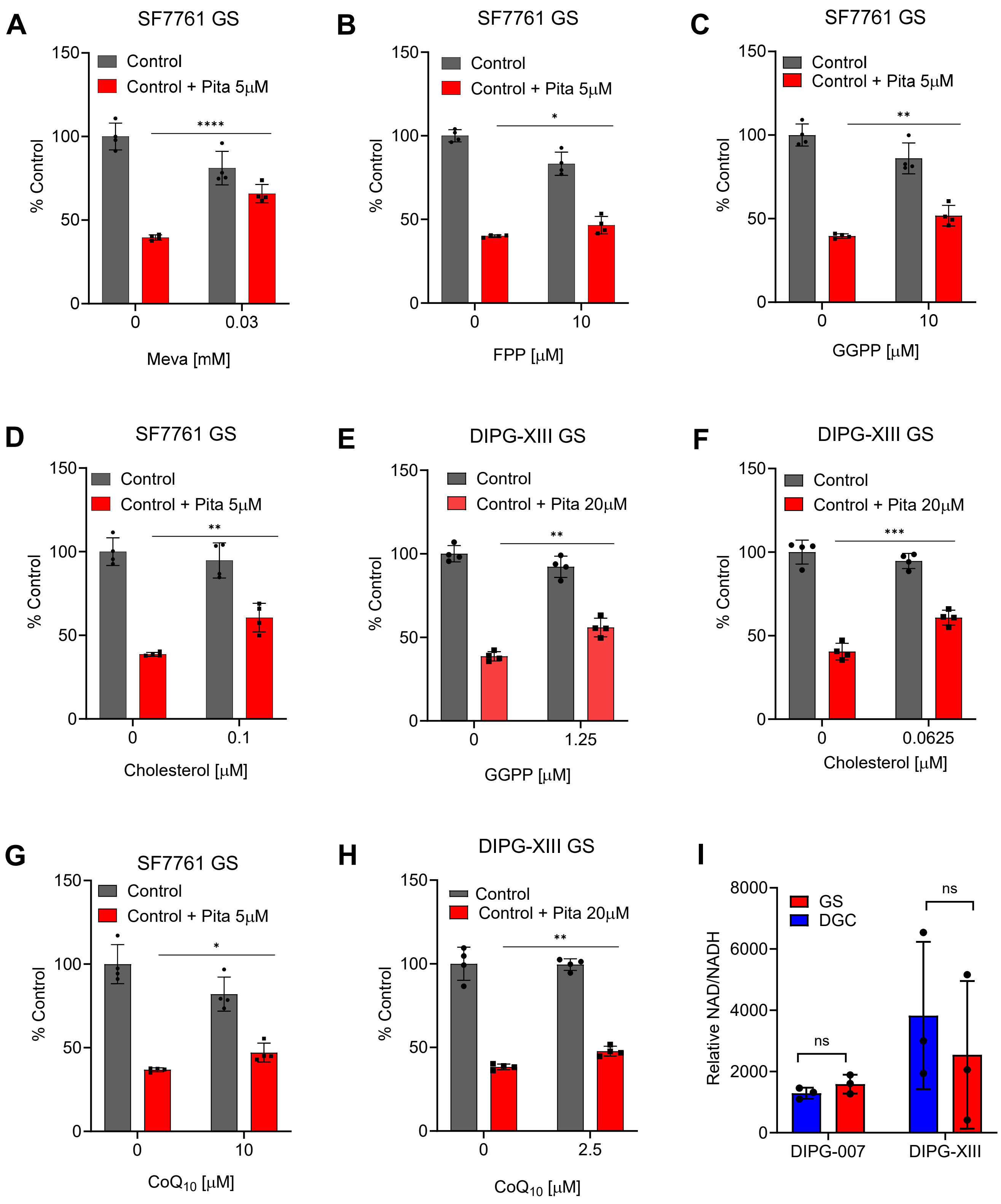

A
SF7761 GS
Control
Control + Pita 5μM
% Control
****
Meva [mM]
0
0.03
B
SF7761 GS
Control
Control + Pita 5μM
*
FPP [μM]
0
10
C
SF7761 GS
Control
Control + Pita 5μM
**
GGPP [μM]
0
10
D
SF7761 GS
Control
Control + Pita 5μM
**
Cholesterol [μM]
0
0.1
E
DIPG-XIII GS
Control
Control + Pita 20μM
**
GGPP [μM]
0
1.25
F
DIPG-XIII GS
Control
Control + Pita 20μM
***
Cholesterol [μM]
0
0.0625
G
SF7761 GS
Control
Control + Pita 5μM
*
CoQ10 [μM]
0
10
H
DIPG-XIII GS
Control
Control + Pita 20μM
**
CoQ10 [μM]
0
2.5
I
GS
DGC
Relative NAD/NADH
ns
ns
DIPG-007
DIPG-XIII

Supplementary Figure 12: Dependency of DIPG gliomaspheres (GS) on cholesterol biosynthesis. A-D) Cell viability of SF7761 GS following treatment with vehicle (0.4% DMSO) or Pitavastatin (Pita) with or without co-treatment with mevalonate (Meva), farnesyl pyrophosphate (FPP), geranylgeranyl pyrophosphate (GGPP), or cholesterol, at the indicated concentrations **E,F)** Cell viability of DIPG-XIII GS following treatment with vehicle (0.4% DMSO) or Pita with or without co-treatment with GGPP or cholesterol, at the indicated concentrations **G,H)** Cell viability of **G)** SF7761 GS and **H)** DIPG-XIII GS following treatment with vehicle (0.4% DMSO) or Pita with or without co-treatment with coenzyme Q_{10} (CoQ_{10}), at the indicated concentrations **A-H)** Cell viability was assayed at 72 hours post-treatment using Cell Titer-Glo 3D with results expressed as percent of vehicle control and error bars representing mean ± SD from three biological replicates; ns = not significant, * $p < 0.05$; ** $p < 0.01$; *** $p < 0.001$; **** $p < 0.0001$ by Unpaired T-test. **I)** Relative NAD/NADH levels in GS vs. differentiated glioma cell (DGC) DIPG-007 and DIPG-XIII, as determined by mass spectrometry. Results are expressed as mean ± SD from three biological replicates. For all panels, red indicates GS; blue, DGC.

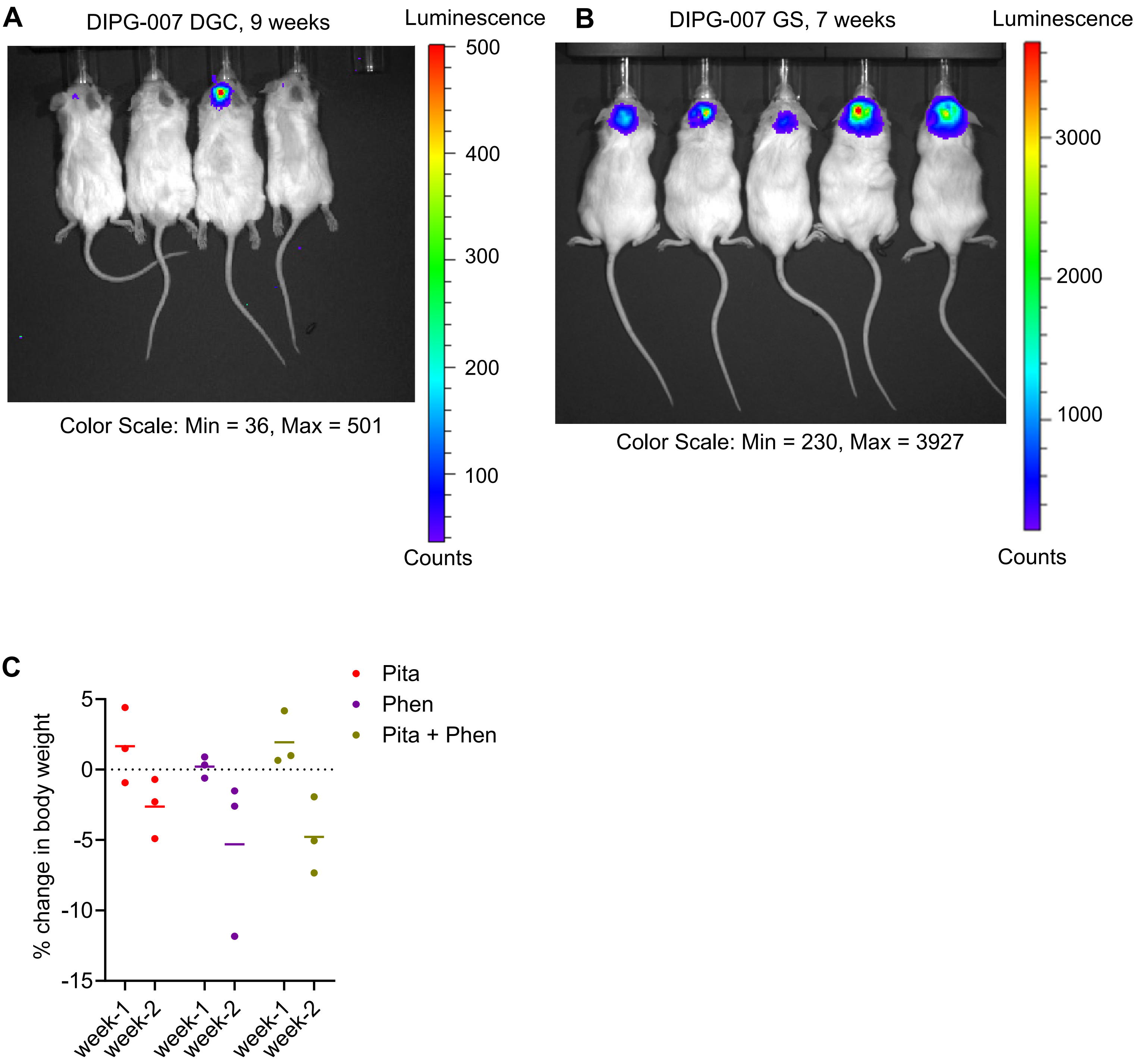
A
DIPG-007 DGC, 9 weeks
Luminescence
500
400
300
200
100
Counts
Color Scale: Min = 36, Max = 501
B
DIPG-007 GS, 7 weeks
Luminescence
3000
2000
1000
Counts
Color Scale: Min = 230, Max = 3927
C
Pita
Phen
Pita + Phen
% change in body weight
5
0
-5
-10
-15
week-1
week-2
week-1
week-2
week-1
week-2

Supplementary Figure 13: Establishment of tumor growth and treatment parameters. **A)** DIPG-007 differentiated glioma cells (DGC) or **B)** gliomaspheres (GS) were orthotopically implanted into the pons of NSG mice. At 9- and 7-weeks post implantation, respectively, tumors were visualized using bioluminescence imaging (BLI). Minimum (Min) and maximum (Max) luminescence values are presented below the images, with color scales ranging from 36 (purple) to 501 (red) for DGC and 230 (purple) to 3927 (red) for GS. **C)** Percent change in body weight was assessed using a 2-week dosing study with Pitavastatin (Pita), Phenformin (Phen), or the combination (Pita + Phen) of both drugs to assess safety and tolerability. Three mice were included in each arm. Mice were dosed on day 1 (Pita,10mg/kg; Phen 50mg/kg) and day 8 (Pita, 20mg/kg; Phen,100mg/kg). Each point represents one mouse, n=1.

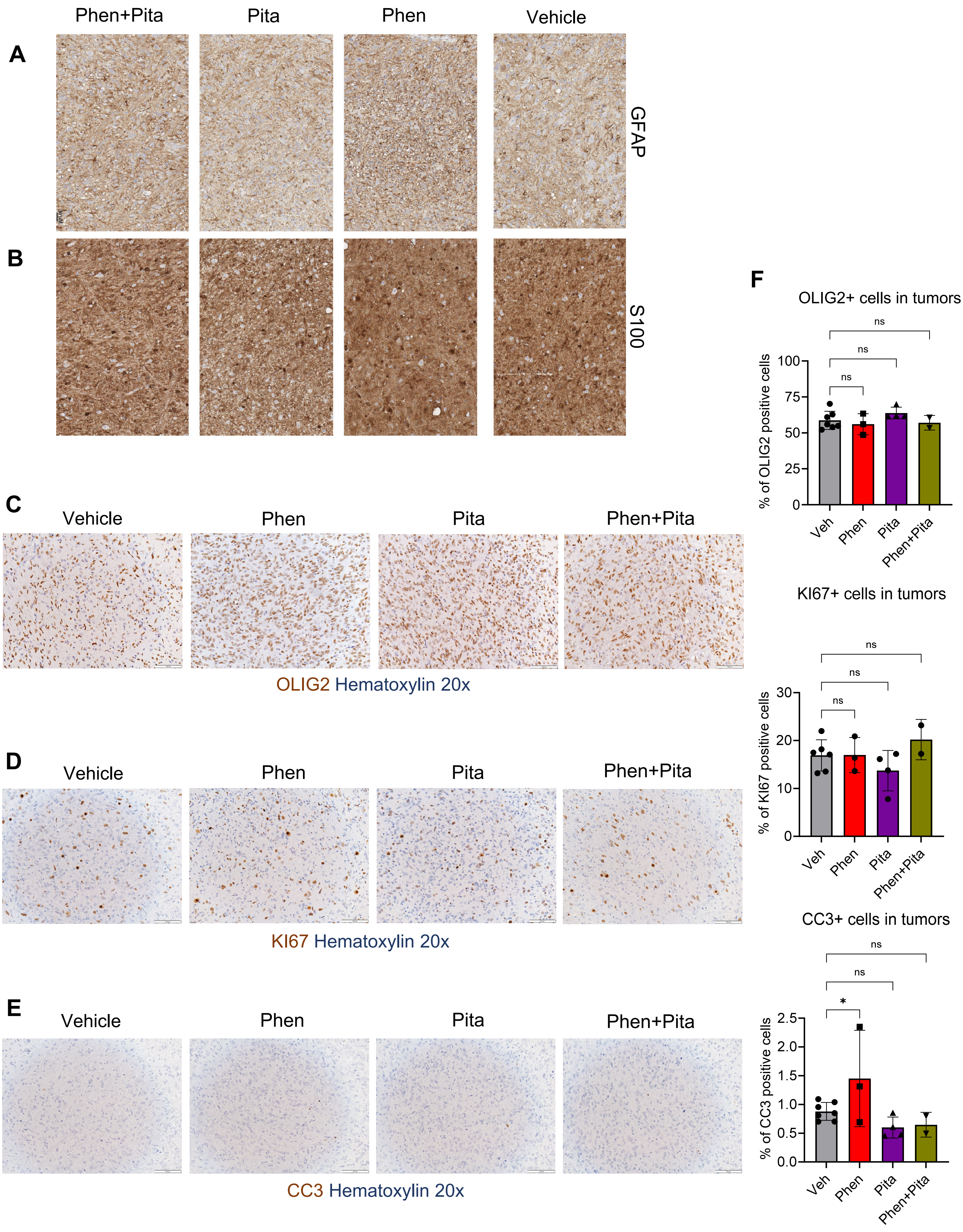

Phen+Pita
Pita
Phen
Vehicle
A
GFAP
B
S100
C
Vehicle
Phen
Pita
Phen+Pita
OLIG2 Hematoxylin 20x
D
Vehicle
Phen
Pita
Phen+Pita
KI67 Hematoxylin 20x
E
Vehicle
Phen
Pita
Phen+Pita
CC3 Hematoxylin 20x
F
OLIG2+ cells in tumors
% of OLIG2 positive cells
ns
ns
ns
Veh
Phen
Pita
Phen+Pita
KI67+ cells in tumors
% of KI67 positive cells
ns
ns
ns
Veh
Phen
Pita
Phen+Pita
CC3+ cells in tumors
% of CC3 positive cells
ns
ns
*
Veh
Phen
Pita
Phen+Pita

Supplementary Figure 14: Histological analysis of DIPG-007 gliomasphere (GS) tumors. Representative images of Figure 7 endpoint tumors that were fixed and stained for the differentiation/stemness markers **A)** glial fibrillary acidic protein (GFAP), **B)** S100, and **C)** oligodendrocyte transcription factor 2 (OLIG2), the proliferation marker **D)** KI67, or the apoptotic marker **E)** cleaved caspase 3 (CC3). Protein expression is indicated in brown and sections were counterstained with hematoxylin in blue. Images for panels A and B were captured at 40X from scanned slides using the Aperio ImageScope, with scale bars indicating 30um, n=3. For OLIG2, KI67 and CC3 (C-E), multiple tumors were stained (scale bars indicate 100um) and quantitated using QuPath software **(F)**. Each point represents the average of 3 images per tumor, with error bars representing mean ± SD; ns = not statistically significant; * $p < 0.05$ by one-way ANOVA statistical test, Tukey's multiple comparisons test.
