# Supplementary material for: Therapeutic targeting of differentiation-state dependent metabolic vulnerabilities in diffuse midline glioma
Source: Nat Commun. 2024 Oct 17;15:8983. doi: 10.1038/s41467-024-52973-4 (PMC11487135; doi:10.1038/s41467-024-52973-4)
Supplement: Supplementary file 3 — Description of Additional Supplementary Information [file 41467_2024_52973_MOESM3_ESM.docx]

**Description of Additional Supplementary Files**

File Name: Supplementary Data 1

Description: (STR Profiling). Small tandem repeat (STR) profiling of differentiated glioma cell (DGC) and GS DIPG-007, SF7761, and DIPG-XIII cell lines.

File Name: Supplementary Data 2

Description: (Metabolomics). Raw, unprocessed ion counts for the targeted metabolomics data presented in Figure 2 and Supplementary Figures 5 and 6.

File Name: Supplementary Data 3

Description: (Tracing tables). Raw, unprocessed ion counts for the isotope tracing metabolomics data presented in Figure 5 and Supplementary Figures 10 and 11.
