# Supplementary material for: Therapeutic targeting of differentiation-state dependent metabolic vulnerabilities in diffuse midline glioma
Source: Nat Commun. 2024 Oct 17;15:8983. doi: 10.1038/s41467-024-52973-4 (PMC11487135; doi:10.1038/s41467-024-52973-4)
Supplement: Supplementary file 7 — Reporting Summary [file 41467_2024_52973_MOESM7_ESM.pdf]

nature portfolio

Corresponding author(s): Costas Lyssiotis

Last updated by author(s): Feb 22, 2024

Reporting Summary

Nature Portfolio wishes to improve the reproducibility of the work that we publish. This form provides structure for consistency and transparency in reporting. For further information on Nature Portfolio policies, see our Editorial Policies and the Editorial Policy Checklist.

Statistics

For all statistical analyses, confirm that the following items are present in the figure legend, table legend, main text, or Methods section.

n/a	Confirmed	
☐	☒	The exact sample size (*n*) for each experimental group/condition, given as a discrete number and unit of measurement
☐	☒	A statement on whether measurements were taken from distinct samples or whether the same sample was measured repeatedly
☐	☒	The statistical test(s) used AND whether they are one- or two-sided<br>*Only common tests should be described solely by name; describe more complex techniques in the Methods section.*
☒	☐	A description of all covariates tested
☐	☒	A description of any assumptions or corrections, such as tests of normality and adjustment for multiple comparisons
☐	☒	A full description of the statistical parameters including central tendency (e.g. means) or other basic estimates (e.g. regression coefficient) AND variation (e.g. standard deviation) or associated estimates of uncertainty (e.g. confidence intervals)
☐	☒	For null hypothesis testing, the test statistic (e.g. *F*, *t*, *r*) with confidence intervals, effect sizes, degrees of freedom and *P* value noted<br>*Give P values as exact values whenever suitable.*
☒	☐	For Bayesian analysis, information on the choice of priors and Markov chain Monte Carlo settings
☒	☐	For hierarchical and complex designs, identification of the appropriate level for tests and full reporting of outcomes
☒	☐	Estimates of effect sizes (e.g. Cohen's *d*, Pearson's *r*), indicating how they were calculated

Our web collection on statistics for biologists contains articles on many of the points above.

Software and code

Policy information about availability of computer code

Data collection	Protein lysate concentrations were measured using SoftMax Pro 6.5.1 on a SpectraMax M3 plate reader.<br>To detect lipid ROS, C11-BODIPY signal was captured with the FITC channel on a ZE5 Cell analyzer.<br>Seahorse data was collected on a XF-96 Extracellular Flux Analyzer and analysis was performed using the Wave 2.3 software.<br>Agilent Masshunter Workstation Software LC/MS Data Acquisition for 6400 Series Triple Quadrupole MS with Version B.08.02 was used for compound optimization, calibration, and data acquisition of metabolomics samples.<br>Lipid profiling was conducted using a Vanquish UHPLC system with an Orbitrap Fusion Lumos Tribrid™ mass spectrometer.<br>RNA-Seq reads were trimmed using Trimmomatic v0.3683 and the library qualities were assessed using FastqQC v0.11 for trimmed reads. RSEM v1.3.1 and STAR v2.5.2a were used to generate paired-end alignments and counts.<br>Western blot images were captured with a Bio-Rad ChemiDdoc imager using Image Lab Touch Software, version 2.4.0.03.<br>Bioluminescent imaging software on the IVIS Spectrum In Vivo Imaging System ??<br>Immunohistochemistry slides were imaged with CellSens Standard software using an Olympus BX53F microscope, fitted with an Olympus DP80 digital camera (Olympus Life Science).
Data analysis	Viability assay data was analyzed using GraphPad Prism 8 software.<br>Analysis of lipid ROS data was performed using FlowJo v.10 software.<br>Seahorse analysis was performed using the Wave 2.3 software.<br>The QqQ metabolomics data were pre-processed with Agilent MassHunter Workstation QqQ Quantitative Analysis Software (B0700). Graphs were generated using GraphPad Prism 8.0 software. Heatmaps were generated and data clustered using Morpheus Matrix Visualization and analysis tool (https://software.broadinstitute.org/morpheus). Pathway analyses were conducted using MetaboAnalyst (https://www.metaboanalyst.ca).

Data processing for glucose isotope tracing analyses was performed in Agilent MassHunter Workstation Profinder 10.0 Build 10.0.10062.0. Isotopologue distributions were derived from a compound standard library built in Agilent MassHunter PCDL (Personal Compound and Database Library) v7.0.
Raw lipidomics data was converted into mzML format using Proteowizard mscovert software 81. MS-DIAL software (verion 4.9.221218)82 was used for general lipidomics data analysis including compound identification with LipidBlast which is default library in MS-DIAL.
RNA-Seq Differential gene expression analysis was performed using DESeq2 v1.26.0. Normalized counts were obtained using DESeq2. Differentially expressed genes were analyzed using GSEA (http://software.broadinstitute.org/gsea/index.jsp) using the HALLMARK gene sets.
Western Blot densitometry was performed using Bio-Rad Image Lab software.
Positive immunohistochemical DAB signal was quantified using QuPath software. Graphs were generated and statistics were calculated using GraphPad Prism 8.

For manuscripts utilizing custom algorithms or software that are central to the research but not yet described in published literature, software must be made available to editors and reviewers. We strongly encourage code deposition in a community repository (e.g. GitHub). See the Nature Portfolio guidelines for submitting code & software for further information.

Data

Policy information about availability of data

All manuscripts must include a data availability statement. This statement should provide the following information, where applicable:

- Accession codes, unique identifiers, or web links for publicly available datasets
- A description of any restrictions on data availability
- For clinical datasets or third party data, please ensure that the statement adheres to our policy

Provide your data availability statement here.

Research involving human participants, their data, or biological material

Policy information about studies with human participants or human data. See also policy information about sex, gender (identity/presentation), and sexual orientation and race, ethnicity and racism.

Reporting on sex and gender	Sex and gender were not factored into the analysis.
Reporting on race, ethnicity, or other socially relevant groupings	Race, ethnicity, or other socially relevant groupings were not factored into the analysis.
Population characteristics	The human DIPG and DMG dataset used in this study was mined from Mackay, A. et al. Integrated Molecular Meta-Analysis of 1,000 Pediatric High-Grade and Diffuse Intrinsic Pontine Glioma. Cancer Cell 32, 520-537.e5 (2017).
Recruitment	As this was a mined data analysis, recruitment was not performed in our study.
Ethics oversight	As this was a mined data analysis, ethics oversight was not providing by the University of Michigan Institutional Review Board.

Note that full information on the approval of the study protocol must also be provided in the manuscript.

Field-specific reporting

Please select the one below that is the best fit for your research. If you are not sure, read the appropriate sections before making your selection.

[x] Life sciences   [ ] Behavioural & social sciences   [ ] Ecological, evolutionary & environmental sciences

For a reference copy of the document with all sections, see nature.com/documents/nr-reporting-summary-flat.pdf

Life sciences study design

All studies must disclose on these points even when the disclosure is negative.

Sample size	No sample size calculation was performed. Sample sizes were chosen based on standard experimental group sizes to achieve acceptable power taking into account the increased variability of animal models (3-4 replicates for in vitro experiments, and 8-10 replicates for in vivo experiments).
Data exclusions	No data was excluded from analyses in these studies.
Replication	Experiments were repeated at least once with good replication of results. Data points were comprised of 3 biological replicates for proliferation and viability studies. RNA-Seq and metabolomics studies were run once using samples prepared from 3 biological replicates. Morphology studies and cell proliferation assays for glucose reduction and glutamine deprivation studies were performed on 2 independent wells.
Randomization	For in vivo experiments, animals (including littermates of the same sex) were randomly assigned to control or treatment conditions.
Blinding	Blinding was not possible as the treatments were administered by the same party responsible for data collection.

Reporting for specific materials, systems and methods

We require information from authors about some types of materials, experimental systems and methods used in many studies. Here, indicate whether each material, system or method listed is relevant to your study. If you are not sure if a list item applies to your research, read the appropriate section before selecting a response.

Materials & experimental systems

n/a	Involved in the study
☐	☒ Antibodies
☐	☒ Eukaryotic cell lines
☒	☐ Palaeontology and archaeology
☐	☒ Animals and other organisms
☒	☐ Clinical data
☒	☐ Dual use research of concern
☒	☐ Plants

Methods

n/a	Involved in the study
☒	☐ ChIP-seq
☒	☐ Flow cytometry
☒	☐ MRI-based neuroimaging

Antibodies

Antibodies used

Olig2 (E6G6Q) XP® Rabbit mAb; Cell Signaling Technology; #65915; Western Blotting (1:1000) and Immunohistochemistry (1:100)
PARP Rabbit Polyclonal Antibody; Cell Signaling Technology; #9542; Western Blotting (1:1000)
HSP90 Rabbit Polyclonal Antibody; Cell Signaling Technology; #4874; Western Blotting (1:10,000)
α-Tubulin (11H10) Rabbit mAb; Cell Signaling Technology; #2125; Western Blotting (1:10,000)
Anti-KI67 (Abcam; #ab15580; Immunohistochemistry (1:1000)
Cleaved Caspase-3 (Asp175) (5A1E) Rabbit mAb; Cell Signaling Technology; #9664; Immunohistochemistry (1:100)
anti-GFAP (Dako; #Z0334; Immunohistochemistry (1:1000)
anti-S100 (Dako; #GA50461-2; Immunohistochemistry (1:200)

Validation

All antibodies were validated by their commercial sources as indicated below:
Olig2 (E6G6Q) XP® Rabbit mAb #65915 from Cell Signaling Technologies: Olig2 (E6G6Q) XP® Rabbit mAb recognizes endogenous levels of total Olig2 protein of human, mouse and rat origin and is suitable for Western Blotting, Immunoprecipitation, Immunohistochemistry (Paraffin), and Immunofluorescence (Frozen).
PARP Antibody #9542 from Cell Signaling Technologies: PARP Rabbit Polyclonal Antibody detects endogenous levels of full length PARP1 (116 kDa), as well as the large fragment (89 kDa) of PARP1 resulting from caspase cleavage of human, mouse, rat and monkey origin. The antibody does not cross-react with related proteins or other PARP isoforms. It is suitable for Western Blotting.
HSP90 Antibody #4874 from Cell Signaling Technologies: HSP90 Rabbit Polyclonal Antibody detects endogenous levels of total HSP90 protein, alpha and beta isoforms of human, mouse, rat, monkey, D. melanogaster, and zebrafish. This antibody does not cross-react with other HSPs. It is suitable for Western Blotting and Immunohistochemistry (Paraffin).
α-Tubulin (11H10) Rabbit mAb #2125 from Cell Signaling Technologies: α-Tubulin (11H10) Rabbit mAb detects endogenous levels of total α-tubulin protein, and does not cross-react with recombinant β-tubulin. It is reactive in human, mouse, rat, monkey, D. melanogaster, zebrafish, bovine and pig and is suitable for Western Blotting, Immunohistochemistry (Paraffin), Immunofluorescence (Immunocytochemistry), and Flow Cytometry (Fixed/Permeabilized).
Cleaved Caspase-3 (Asp175) (5A1E) Rabbit mAb #9664 from Cell Signaling Technologies: Cleaved Caspase-3 (Asp175) (5A1E) Rabbit mAb detects endogenous levels of the large fragment (17/19 kDa) of activated caspase-3 resulting from cleavage adjacent to Asp175. This antibody does not recognize full-length caspase-3 or other cleaved caspases. Non-specific labeling may be observed by immunofluorescence in specific sub-types of healthy cells in fixed-frozen tissues (e.g. pancreatic alpha-cells). Cytoplasmic background may be observed in human and monkey samples. It is reactive in human, mouse, rat and monkey and is suitable for Western Blotting, Immunoprecipitation, Immunohistochemistry (Paraffin), Immunofluorescence (Immunocytochemistry) and Flow Cytometry (Fixed/Permeabilized).
Anti-Ki67 antibody (ab15580) from Abcam: Rabbit polyclonal to Ki67 is reactive in human and mouse and is suitable for Immunohistochemistry (Paraffin) and Immunocytochemistry/Immunofluorescence. It has been KO validated.
GFAP, Agilent (DAKO), Catalog #Z0334, Rabbit pAb:
S100, Agilent (DAKO), Catalog #A50461-2, Rabbit pAb:

Eukaryotic cell lines

Policy information about cell lines and Sex and Gender in Research

Cell line source(s)	HSJD-DIPG-007 (referred to as DIPG-007, H3.3K27M) was obtained from Dr. Rintaro Hashizume, Northwestern University; RRID: CVCL_VU70. SU-DIPG-XIII (referred to as DIPG-XIII, H3.3K27M) was obtained from Dr. Michelle Monje, Stanford University; RRID: CVCL_6948. SF7761 (H3.3K27M) was purchased from Millipore Sigma (#Cat.no. SCC126).<br>PPK cells were generated as an In Utero Electroporation (IUE) murine model of H3K27M glioma. IUE was performed using sterile technique on isoflurane/oxygen-anesthetized pregnant C57BL/6 or CD1 females at E13.5. Tumors were generated with lateral ventricle (forebrain) introduction of plasmids: (1) PB-CAG-DNp53-Ires-Luciferase (dominant negative TP53), (2) PB-CAG-PdgfraD824V-Ires-eGFP (PDGFRA D842V), and (3) PB-CAG-H3.3 K27M-Ires-eGFP (H3K27M).
Authentication	Human cell lines were authenticated by the Arizona Genetics Core, a GLP qualified laboratory that provides autosomal STR profiles suitable for verification using reference databases (such as ATCC, DSMZ, JCRB).
Mycoplasma contamination	Cell lines regularly tested fnegative for mycoplasma contamination using the LONZA MycoAlert PLUS Mycoplasma Detection Kit, cat# LT07-710.
Commonly misidentified lines (See ICLAC register)	None used

Animals and other research organisms

Policy information about studies involving animals; ARRIVE guidelines recommended for reporting animal research, and Sex and Gender in Research

Laboratory animals	NOD-SCID-IL2R gamma chain-deficient (NSG) mice that were 8–10 weeks of age were used in these studies. Animals were housed and cared for according to standard University of Michigan guidelines with free access to food and water and kept at constant ambient temperature with a 12-hour light cycle.
Wild animals	None used
Reporting on sex	Males and females were used equally. Animals, including littermates of the same sex, were randomly assigned to control or treatment conditions.
Field-collected samples	None used
Ethics oversight	All animal studies were performed in accordance with the guidelines of the University of Michigan Institutional Animal Care and Use Committee under our approved animal protocol (PRO00008865).

Note that full information on the approval of the study protocol must also be provided in the manuscript.

Plants

Seed stocks	*Report on the source of all seed stocks or other plant material used. If applicable, state the seed stock centre and catalogue number. If plant specimens were collected from the field, describe the collection location, date and sampling procedures.*
Novel plant genotypes	*Describe the methods by which all novel plant genotypes were produced. This includes those generated by transgenic approaches, gene editing, chemical/radiation-based mutagenesis and hybridization. For transgenic lines, describe the transformation method, the number of independent lines analyzed and the generation upon which experiments were performed. For gene-edited lines, describe the editor used, the endogenous sequence targeted for editing, the targeting guide RNA sequence (if applicable) and how the editor was applied.*
Authentication	*Describe any authentication procedures for each seed stock used or novel genotype generated. Describe any experiments used to assess the effect of a mutation and, where applicable, how potential secondary effects (e.g. second site T-DNA insertions, mosiacism, off-target gene editing) were examined.*
